# Pediatric tumours: pseudotumour or a tumour?

**DOI:** 10.1186/1470-7330-14-S1-O14

**Published:** 2014-10-09

**Authors:** Thierry AGM Huisman

**Affiliations:** 1 Division of Pediatric Radiology and Pediatric Neuroradiology, Johns Hopkins Hospital, 1800 Orleans Street, Suite 4174, Baltimore, MD 21287-0842, USA

## 

Differentiation between tumours and pseudo-tumours is essential for planning adequate treatment and for estimating outcome and future prognosis. Per definition, tumour and pseudo-tumours are lesions that look alike on ultrasound (US), computer tomography (CT) or magnetic resonance imaging (MRI) studies. Misinterpretation may lead to a significant delay of adequate treatment for malignant tumours or on the contrary may result in over-treatment of a tumour-like, benign lesion. The essential question is how does a radiologist reliably differentiate between both entities? Typically, he will use anatomical imaging studies like US, CT or MRI. A tumour is suspected when a focal density or signal alteration is seen displacing or infiltrating adjacent structures with or without a matching contrast enhancement and possibly surrounded by vasogenic edema. Unfortunately, many pseudo-tumours including abscesses, resolving hematomas, vascular malformations, benign cysts, and even metabolic disorders may have similar imaging features. In addition, a frequent, difficult question is the differentiation between postsurgical changes and residual tumour after recent tumour surgery or between radiation induced lesions and recurrent or residual tumour. The recent development of functional MRI sequences like diffusion tensor imaging (DTI), perfusion weighted imaging (PWI), ^1^H magnetic resonance spectroscopy (MRS), susceptibility weighted imaging (SWI) and dynamic contrast enhanced MRI sequences facilitates the differentiation between tumours and pseudo-tumours.

It is impossible to present a complete list of pseudo-tumours, however the current presentation will discuss how a multidisciplinary, multi-imaging and multi-sequence approach may facilitate differentiation between tumors and pseudo-tumours.

